# Outcomes of Left Atrial Appendage Closure in Hemodialysis Patients With Atrial Fibrillation: The OCEAN-LAAC Registry

**DOI:** 10.1016/j.jacasi.2024.10.022

**Published:** 2025-01-21

**Authors:** Shuhei Tanaka, Teruhiko Imamura, Nobuyuki Fukuda, Hiroshi Ueno, Koichiro Kinugawa, Mitsuru Sago, Ryuki Chatani, Daisuke Hachinohe, Toru Naganuma, Yohei Ohno, Tomoyuki Tani, Hideharu Okamatsu, Kazuki Mizutani, Yusuke Watanabe, Masaki Izumo, Mike Saji, Shingo Mizuno, Shunsuke Kubo, Shinichi Shirai, Masaki Nakashima, Masahiko Asami, Masanori Yamamoto, Kentaro Hayashida, OCEAN-LAAC Investigators

**Affiliations:** a Second Department of Internal Medicine, University of Toyama, Toyama, Japan; b Department of Cardiology, Toyohashi Heart Center, Aichi, Japan; c Department of Cardiology, Kurashiki Central Hospital, Okayama, Japan; d Division of Cardiovascular Medicine, Sapporo Heart Center, Sapporo Cardio Vascular Clinic, Sapporo, Japan; e Department of Cardiology, New Tokyo Hospital, Chiba, Japan; f Department of Cardiology, Tokai University School of Medicine, Kanagawa, Japan; g Department of Cardiology, Sapporo East Tokushukai Hospital, Hokkaido, Japan; h Department of Cardiology, Saiseikai Kumamoto Hospital, Kumamoto, Japan; i Department of Cardiology, Kinki University school of Medicine, Osaka, Japan; j Department of Cardiology, Teikyo University School of Medicine, Tokyo, Japan; k Department of Cardiology, St. Marianna University School of Medicine, Kanagawa, Japan; l Department of Cardiology, Sakakibara Heart Institute, Tokyo, Japan; m Division of Cardiovascular medicine, Toho University Faculty of Medicine; n Department of Cardiology, Shonan Kamakura General Hospital, Kanagawa, Japan; o Department of Cardiology, Kokura Memorial Hospital, Fukuoka, Japan; p Department of Cardiology, Sendai Kousei Hospital, Miyagi, Japan; q Division of Cardiology, Mitsui Memorial Hospital, Tokyo, Japan; r Department of Cardiology, Nagoya Heart Center, Aichi, Japan; s Department of Cardiology, Gifu Heart Center, Gifu, Japan; t Department of Cardiology, Keio University School of Medicine, Tokyo, Japan

**Keywords:** anticoagulation, hemodialysis, ischemic stroke, left atrial appendage closure, nonvalvular atrial fibrillation

## Abstract

**Background:**

Left atrial appendage closure (LAAC) has demonstrated favorable outcomes as an alternative to permanent anticoagulation in patients with nonvalvular atrial fibrillation (NVAF). In hemodialysis (HD) patients with NVAF, anticoagulation increases bleeding complications, with inconsistent benefits in stroke prevention.

**Objectives:**

This study aimed to clarify the benefit of LAAC for HD patients.

**Methods:**

Consecutive patients who underwent LAAC, as extracted from the Japanese multicenter registry, were eligible. When comparing HD and non-HD patients, perioperative events, including LAAC procedure success rates and the incidence of death, bleeding events, and ischemic stroke events, were analyzed.

**Results:**

Among 1,464 patients (mean age 77.1 ± 7.6 years, mean CHA_2_DS_2_-VASc score 4.9 ± 1.5, mean HAS-BLED score 3.1 ± 1.0), 172 were HD patients. The HD group had higher HAS-BLED scores, whereas more patients in the non-HD group had a history of Bleeding Academic Research Consortium type 3 bleeding. Device implantation success was 97.3% (95% CI: 96.3%-98.0%) (HD group; 97.1% [167 of 172], non-HD group; 97.3% [1,257 of 1,292]; *P =* 0.88). There were no in-hospital deaths, and perioperative complications were rare and did not differ between the 2 groups. The median follow-up period was 367 days (Q1-Q3: 242-422 days). The ischemic stroke rate following LAAC in the HD group was 1.1% (95% CI: 0.3%-1.9%) per 100 patient-years, comparable to the non-HD group.

**Conclusions:**

LAAC is feasible for HD patients and achieves results comparable to those in non-HD patients. Further research is necessary to determine the effectiveness of LAAC in preventing stroke in HD patients.

Left atrial appendage closure (LAAC) has emerged as a widely adopted non-pharmacologic alternative to anticoagulation therapy for patients with non-valvular atrial fibrillation (NVAF) who are contraindicated for long-term anticoagulation therapy caused by a variety of reasons. Based on evidence from recently conducted large-scale randomized clinical trials such as the PROTECT AF (Watchman Left Atrial Appendage Closure Technology for Embolic Protection in Patients With Atrial Fibrillation)^1^ and PREVAIL (Evaluation of the WATCHMAN Left Atrial Appendage (LAA) Closure Device in Patients With Atrial Fibrillation Versus Long Term Warfarin Therapy) trials,^2^ which demonstrated a reduction in long-term hemorrhagic events during therapeutic period over anticoagulation therapy alone in carefully selected cohorts, LAAC using the WATCHMAN system (Boston Scientific) received U.S Food and Drug Administration approval in 2015. In Japan, following the SALUTE (A Study to Evaluate the Safety and Effectiveness of the Left Atrial Appendage Closure Therapy Using BSJ003W) trial,^3^ LAAC was reimbursed by insurance in 2019. Subsequent real-world observational study has also demonstrated favorable outcome for LAAC.^4^ In the PINNACLE FLX (Protection Against Embolism for Nonvalvular AF Patients: Investigational Device Evaluation of the Watchman FLX LAA Closure Technology) trial,^5^ the introduction of next-generation devices further improved procedural safety, and favorable clinical outcomes after LAAC using FLX (the newer device) were demonstrated even in comparison with direct oral anticoagulation (OAC).

In Japan, although OAC for hemodialysis (HD) patients is limited to warfarin, even warfarin is generally not recommended according to the guidelines of the Japanese society for dialysis therapy because of its increased risk of bleeding. Therefore, HD patients with NVAF rarely receive OAC. On the other hand, it has been reported that the prevalence of ischemic stroke is significantly higher in HD patients with NVAF compared with non-HD patients with NVAF.^6^ However, there is limited evidence that warfarin reduces the incidence of ischemic stroke, rather than increasing bleeding events.^7^

Although LAAC is a therapeutic option for preventing cardiogenic stroke or systemic embolization in HD patients with NVAF for whom anticoagulation is generally contraindicated in Japan, there is currently a lack of comprehensive reports. Therefore, in this study, we analyzed the safety and efficacy of LAAC in HD patients compared with non-HD patients using a large-scale Japanese multicenter registry data.

## Methods

### Study design and patient population

The OCEAN (Optimized CathEter vAlvular iNtervention)-LAAC registry, registered with the University Hospital Medical Information Network (UMIN000038498), is an ongoing, prospective, investigator-initiated, multicenter observational registry of patients with NVAF undergoing percutaneous LAAC in Japan. Twenty centers in Japan participate in the registry. This study was approved by the Institutional Review Board of Keio University Hospital Clinical and Translational Research Center at the lead institution and the ethics committees of each participating center. The registry management was conducted in accordance with the Declaration of Helsinki. All participants provided informed consent before enrollment in this registry. The current data were obtained from patients who underwent LAAC from September 2019 to December 2022. During this period, the generation-2.5 device (the previous device) was used until May 2021. Afterward, the newer device received clinical approval and was introduced as the new-generation device. The patient backgrounds and the periprocedural and early to midterm outcomes were compared between the HD and non-HD groups.

### Indication of LAAC

LAAC was considered as an alternative to long-term anticoagulation therapy for NVAF patients who were at high-risk of stroke or systemic embolism based on their CHADS_2_ score or CHA_2_DS_2_-VASc score. Eligible patients should also have a high bleeding risk satisfying at least 1 of the following conditions: 1) HAS-BLED score equal or above 3; 2) a history of multiple instances of trauma from falls requiring treatment; 3) a history of cerebral amyloid angiopathy; 4) the necessity of combination of dual-antiplatelet therapy (DAPT) for over 1 year; and 5) a history of major bleeding classified as Bleeding Academic Research Consortium type 3.^8^

The exclusion criteria for LAAC were as follows: 1) thrombus in heart, especially left atrium; 2) a history of repair therapy for atrial septal defect or patent foramen ovale; 3) unsuitable morphology for LAAC; 4) contraindications for transesophageal echocardiography; and 5) contraindications for anticoagulation therapy or any antiplatelet therapy.

### The Procedure of transcatheter LAAC and definition of the procedural outcome

LAAC was performed under general anesthesia, transesophageal echocardiography (TEE) guidance via femoral vein, and transseptal approach according to the standard procedure by the board-certificated interventionalists. The previous and the newer device were utilized in LAAC, with their sizes selected to fit the anatomy and size of each LAA.

Device success was defined by the precise deployment and appropriate positioning of the device, while technical success was achieved as complete closure of the LAA with a peridevice leakage of <5 mm and the absence of device-related complications. Procedural success required the absence of procedural-related complications, in addition to technical success.^9^

### The regimen of antithrombotic therapy

According to the attached document and the previous literature,^1^^,^^2^ it is recommended to add low-dose aspirin after LAAC to OAC, especially warfarin. If the 45-day imaging documented the depth of peridevice flow was <5 mm and no visible device-related thrombosis (DRT), OAC was discontinued and added clopidogrel. Finally, clopidogrel was discontinued 6 months after LAAC.

However, in real-world practice, the regimen of antithrombotic therapy after LAAC, especially whether to add antiplatelet therapy (APT) after the procedure and DAPT period following the first follow-up, and so on, was determined by each facility.

### Study outcomes

Postprocedural in-hospital data were collected, such as adverse events including all-cause mortality, all-cause bleeding, and any stroke; and other procedure-related complications such as access site complication, TEE-associated complication, pulmonary complication, device embolization, and requirement of iatrogenic atrial septal defect closure.

Clinical follow-up after LAAC were scheduled at 45 to 90 days and 1 year. Follow-up imaging data was generally obtained through TEE or contrast-enhanced computed tomography. However, in this study, only the results of TEE were analyzed. Follow-up data were collected including all-cause death, cardiovascular death, all-cause hospitalization, major or any bleeding, ischemic stroke, and DRT. Major bleeding was defined as Bleeding Academic Research Consortium type 3 or 5.

### Statistical analyses

Statistical analyses were performed with JMP Pro 16 (SAS Institute Inc). Two-sided *P* values <0.05 were considered statistically significant. Continuous variables were expressed as the mean ± SD. Categorical variables were expressed as numbers and percentages. Wilcoxon-signed rank test was performed to compare the coupled data. Continuous variables were compared by Student’s *t*-test or the Mann-Whitney *U* test, depending on their distribution. The Pearson’s chi-square test or Fisher exact test was applied for categorical variables.

Cox proportional HR regression analysis was performed to investigate the impact of maintenance hemodialysis on outcomes following LAAC. The time-dependent covariates were not statistically significant (*P >* 0.05), indicating that the proportional hazards assumption was satisfied. Kaplan-Meier analysis and log-rank tests were conducted to compare clinical outcomes stratified with or without maintenance hemodialysis.

## Results

### Baseline characteristics

A total of 1,464 patients were enrolled in this study. Among them, there were 172 patients on maintenance HD. Baseline characteristics of the entire cohort were presented in Table 1. Mean age was 77.1 ± 7.6 years, and 68% (990 of 1,464) were men. The percentage of patients with a history of ischemic stroke and major bleeding were 38% (549 of 1,464) and 58% (850 of 1,464), respectively. CHA_2_DS_2_-VASc score was 4.9 ± 1.5 and HAS-BLED score was 3.1 ± 1.0, respectively.

**Table 1:** Baseline Characteristics

	All (N = 1,464)	Non-HD (n = 1,292)	HD (n = 172)	*P* Value
Age, y	77.1 ± 7.6	77.5 ± 7.5	74.2 ± 8.2	<0.0001
Male	990 (67.6)	864 (66.9)	126 (73.3)	0.0928
Body surface area, m^2^	1.62 ± 0.20	1.62 ± 0.20	1.62 ± 0.20	0.8902
Clinical frailty scale	3.1 ± 1.2	3.1 ± 1.2	3.3 ± 1.3	0.0192
Sinus rhythm on admission	468 (32.0)	399 (30.9)	69 (40.1)	0.0147
History of catheter ablation for AF	353 (24.1)	312 (24.1)	41 (23.8)	0.9285
Systolic blood pressure, mm Hg	126 ± 19	126 ± 19	126 ± 22	0.6886
Diastolic blood pressure, mm Hg	72 ± 13	72 ± 13	70 ± 12	0.0306
Heart rate, beats/min	73 ± 14	72 ± 14	74 ± 14	0.2127
Congestive heart failure	718 (49.0)	641 (49.6)	77 (44.8)	0.2324
Hypertension	1,192 (81.4)	1,049 (81.1)	143 (83.1)	0.5373
Uncontrolled hypertension	120 (8.2)	94 (7.3)	26 (15.1)	0.0004
Diabetes mellitus	514 (35.1)	436 (33.7)	78 (45.3)	0.0027
Dyslipidemia	615 (42.0)	536 (41.5)	79 (45.9)	0.2673
Vascular disease	393 (26.8)	326 (25.2)	67 (39.0)	0.0001
Abnormal renal function	226 (15.4)	54 (4.2)	172 (100)	<0.0001
Abnormal liver function	64 (4.4)	54 (4.2)	10 (5.8)	0.3247
Alcohol	321 (21.9)	298 (23.1)	23 (13.4)	0.0039
Bleeding	904 (61.7)	831 (64.3)	73 (42.4)	0.0039
Any stroke	645 (44.1)	568 (44.0)	77 (44.8)	0.7966
Ischemic stroke	549 (37.5)	481 (37.2)	68 (40.0)	0.5573
Transient ischemic attack	65 (4.4)	54 (4.2)	11 (6.4)	0.1851
Hemorrhagic stroke	169 (11.5)	154 (11.9)	15 (8.7)	0.2175
Thromboembolic event	364 (24.9)	320 (24.8)	44 (25.6)	0.8166
History of LAA thrombi	69 (4.7)	65 (5.0)	4 (2.3)	0.1158
History of major bleeding	850 (58.1)	791 (61.2)	59 (34.3)	<0.0001
CHADS_2_ score	3.2 ± 1.3	3.3 ± 1.3	3.2 ± 1.4	0.4342
CHA_2_DS_2_-VASc score	4.9 ± 1.5	4.9 ± 1.5	4.8 ± 1.6	0.5005
HAS-BLED score	3.1 ± 1.0	3.0 ± 1.0	3.9 ± 1.1	<0.0001
Hemoglobin, g/dL	12.3 ± 2.1	12.4 ± 2.1	11.3 ± 1.6	<0.0001
Albumin, g/dL	3.85 ± 0.46	3.9 ± 0.4	3.5 ± 0.5	<0.0001
Creatinine, mg/dL	1.77 ± 2.00	1.16 ± 0.61	6.35 ± 2.74	<0.0001
BNP, pg/mL	255.2 ± 402.6	205 ± 247	663 ± 898	<0.0001
NT-proBNP, pg/mL	3,651 ± 8,545	1,564 ± 2,680	20,220 ± 17,007	<0.0001
Prothrombin time, s	16.5 ± 5.6	1.43 ± 0.50	1.60 ± 0.56	<0.0001
Activated partial thromboplastin time, s	37.5 ± 13.6	37.2 ± 12.8	39.4 ± 19.2	0.0878
D-dimer, ng/mL	1.88 ± 2.12	1.05 ± 1.76	3.20 ± 6.56	<0.0001
LA dimension, mm	45.4 ± 8.4	45.3 ± 8.6	46.2 ± 7.0	0.1889
LA volume, mL	97.1 ± 44.6	96.3 ± 45.0	103.3 ± 41.4	0.1239
LVDd, mm	47.4 ± 6.9	47.1 ± 6.8	49.6 ± 7.0	<0.0001
LVEF, %	58.7 ± 11.4	59.0 ± 11.3	56.4 ± 11.8	0.0069
Mitral stenosis ≥2	11 (0.8)	11 (0.9)	0 (0)	0.2216
Mitral regurgitation ≥2	187 (13.1)	163 (13.0)	24 (14.2)	0.6471
Aortic stenosis ≥2	52 (3.6)	37 (2.9)	15 (8.7)	0.0001
Aortic regurgitant ≥2	57 (4.0)	46 (3.7)	11 (6.6)	0.0728
Tricuspid regurgitant ≥2	268 (18.8)	246 (19.5)	22 (12.9)	0.0386

Values are mean ± SD or n (%).

BNP = B-type natriuretic peptide; LA = left atrium; LVDd = left ventricular end-diastolic dimension; LVEF = left ventricular ejection fraction; NT-proBNP = N-terminal pro–B-type natriuretic peptide.

### Comparison in baseline characteristics

Baseline characteristics were compared between the HD group and the non-HD group (Table 1). Compared with the non-HD group, the HD group comprised a younger population and exhibited a lower percentage with a history of major bleeding. However, the percentage of a history of ischemic stroke was not significantly different between both groups. The HAS-BLED score was significantly higher in the HD group compared with the non-HD group (3.9 ± 1.1 vs 3.0 ± 1.0; *P* < 0.01), whereas the CHA_2_DS_2_-VASc score was not significantly different between both groups (4.8 ± 1.6 vs 4.9 ± 1.5; *P =* 0.50).

The HD group had more comorbidities: more advanced anemia, hypoalbuminemia, more elevated plasma B-type natriuretic peptide levels, and higher d-dimer levels. Transthoracic echocardiography revealed preserved left ventricular systolic function in both groups, albeit slightly worse in the HD group. The HD group had a higher prevalence of moderate or greater aortic stenosis (8.7% [15 of 172] vs 2.9% [37 of 1,292]; *P* < 0.01).

### Preprocedural TEE findings

Preprocedural TEE findings were summarized in Table 2. LAA function such as LAA emptying velocity, spontaneous echo contrast, and the prevalence of thrombi in LAA did not significantly differ between both groups, whereas the prevalence of complex aortic plaque was higher in the groups of HD. The size of LAA ostium tended to be larger in the HD group, although this difference did not reach statistical significance.

**Table 2:** Periprocedural Data

	All (N = 1,464)	Non-HD (n = 1,292)	HD (n = 172)	*P* Value
Preprocedural TEE findings				
LAA flow velocity, cm/s	30.8 ± 18.9	30.6 ± 18.7	31.9 ± 20.6	0.5786
Complex aortic plaque	52 (6.1)	39 (5.2)	13 (13)	0.0023
SEC, °	1.0 ± 1.1	0.9 ± 1.1	1.1 ± 1.1	0.2013
Dense SEC	174 (13.2)	154 (13.2)	20 (13.3)	0.9775
Thrombus	12 (0.8)	12 (1.0)	0 (0)	0.2044
LAA ostium diameter at 0°, mm	21.6 ± 4.1	21.7 ± 4.1	21.4 ± 4.4	0.4386
LAA ostium diameter at 45°, mm	20.2 ± 4.0	20.3 ± 4.0	19.9 ± 4.0	0.1844
LAA ostium diameter at 90°, mm	20.6 ± 4.2	20.7 ± 4.2	20.3 ± 4.1	0.3053
LAA ostium diameter at 135°, mm	22.5 ± 4.3	22.5 ± 4.3	22.7 ± 4.3	0.5005
LAA depth at 0°, mm	22.7 ± 6.9	22.7 ± 7.0	23.6 ± 6.8	0.1385
LAA depth at 45°, mm	22.6 ± 7.1	22.4 ± 7.1	23.9 ± 6.7	0.0205
LAA depth at 90°, mm	22.8 ± 7.1	22.7 ± 7.0	23.8 ± 7.7	0.0920
LAA depth at 135°, mm	22.4 ± 6.6	22.3 ± 6.6	23.1 ± 6.8	0.1896
Procedural data				
The previous device (generation-2.5)	539 (37.6)	476 (37.6)	63 (37.3)	0.9296
The newer device (FLX)	895 (62.4)	789 (62.4)	106 (62.7)	
Procedure time, min	57.9 ± 32.7	57.7 ± 32.2	59.3 ± 36.1	0.5361
Device size, mm	29.8 ± 3.8	29.8 ± 3.8	29.8 ± 3.8	0.9007
Device size 20/21/24/27/30/31/33/35 mm	19 (1.3)/19 (1.3)/161 (11.2)/362 (25.3)/138 (9.6)/277 (19.3)/187 (13.1)/269 (18.8)	15 (1.2)/18 (1.4)/144 (11.4)/317 (25.1)/123 (9.7)/244 (19.3)/162 (12.8)/240 (19.0)	4 (2.4)/1 (0.6)/17 (10.1)/45 (26.6)/15 (8.9)/33 (19.5)/25 (14.8)/29 (17.2)	0.8301
Number of device requirement	1.2 ± 0.5	1.2 ± 0.5	1.1 ± 0.4	0.1861
Contrast volume, mL	50 (30, 75)	50 (32, 75)	48 (30, 75)	0.0781
Fluoroscopy duration, min	12 (8, 18)	12 (6, 20)	12 (9, 17)	0.8074
Device compression rate at 0°, %	17.4 ± 6.1	17.2 ± 6.1	18.7 ± 5.8	0.005
Device compression rate at 45°, %	17.8 ± 5.9	17.6 ± 5.9	19.0 ± 5.8	0.0067
Device compression rate at 90°, %	17.1 ± 6.3	17.0 ± 6.3	18.2 ± 6.4	0.025
Device compression rate at 135°, %	16.5 ± 5.9	16.5 ± 5.9	16.6 ± 6.1	0.8095
Peri-device leakage >5 mm	0 (0)	0 (0)	0 (0)	1.0000
Device implantation success	1,424 (97.3)	1,257 (97.3)	167 (97.1)	0.881
Technical success	1,421 (97.1)	1,255 (97.1)	166 (96.5)	0.6486
Procedural success	1,381 (94.3)	1,219 (94.4)	162 (94.2)	0.9305
Deep device implantation	50 (3.5)	46 (3.7)	4 (2.5)	0.4265
Requiring RBC transfusion	28 (1.9)	23 (1.8)	5 (2.9)	0.3108
Access site complication	9 (0.6)	8 (0.6)	1 (0.6)	0.9525
TEE associated complication	4 (0.3)	4 (0.3)	0 (0)	0.4649
Pulmonary complication	1 (0.1)	1 (0.1)	0 (0)	0.7151
Acute kidney injury	5 (0.3)	5 (0.4)	0 (0)	0.4138
Pericardial effusion	8 (0.6)	7 (0.5)	1 (0.6)	0.9762
Requiring ASD closure	2 (0.1)	2 (0.2)	0 (0)	0.6055
Device embolization	0 (0)	0 (0)	0 (0)	-
Any stroke during hospitalization	0 (0)	0 (0)	0 (0)	-
All bleeding during hospitalization	12 (0.8)	11 (0.9)	1 (0.6)	0.7019
All cause death during hospitalization	0 (0)	0 (0)	0 (0)	-

Values are mean ± SD, median (25th, 75th percentile), or n (%)

ASD = atrial septal defect; LAA = left atrial appendage; RBC = red blood cell; SEC = spontaneous echo contrast; TEE = transesophageal echocardiography.

### Periprocedural findings

Periprocedural findings were displayed in Table 3. The device use, procedure time, contrast volume, fluoroscopic time, and device size did not significantly differ between the 2 groups, with the most frequently used size being 27 mm. The rate of device implantation success was 97.1% (95% CI: 96.3%-98.0%) and favorable compared with previous reports,^2^^,^^10^ with no significant differences observed between both groups (HD group; 97.1% [167 of 172], non-HD group; 97.3% [1,257 of 1,292]; *P =* 0.88). The HD group exhibited a higher device compression rate, and there were no cases of peridevice leakage in both groups.

**Table 3:** TEE Imaging Data at Short-Term Follow-Up and 1-Year Follow-Up

	All	Non-HD	HD	*P* Value
Short-term follow-up				
The number of cases performed follow-up	1391 (95.7)	1227 (95.8)	164 (95.4)	0.7907
The number of cases performed TEE	953 (66.7)	835 (66.3)	118 (70.2)	0.3052
Peri-device leakage over 3 mm	53 (5.6)	50 (6.0)	3 (2.5)	0.1155
Peri-device leakage over 5 mm	4 (0.4)	4 (0.5)	0 (0)	0.4459
Thrombus in LAA (full/half/little/none)	277 (29.1)/220 (23.1)/164 (17.2)/258 (27.1)	245 (29.3)/185 (22.2)/136 (16.3)/237 (28.4)	32 (27.1)/35 (29.7)/28 (23.7)/21 (17.8)	0.0178
Residual IASD	365 (38.3)	304 (36.4)	61 (51.7)	0.0010
Deep implantation	28 (2.9)	26 (3.1)	2 (1.7)	0.3850
Pericardial effusion	14 (1.5)	12 (1.4)	2 (1.7)	0.7874
Device related thrombus	15 (1.6)	14 (1.7)	1 (0.8)	0.4527
1-year follow-up				
The number of cases performed follow-up	978 (87.4)	864 (88.2)	114 (82.0)	0.0409
The number of cases performed TEE	440 (42.0)	388 (42.4)	52 (39.4)	0.5124
Peri-device leakage 3-5 mm	32 (7.3)	31 (8.0)	1 (1.9)	0.1089
Peri-device leakage over 5 mm	5 (1.1)	5 (1.3)	0 (0)	0.4068
Thrombus in LAA (full/half/little/none)	247 (56.1)/72 (16.4)/33 (7.5)/58 (13.2)	225 (58.0)/60 (15.5)/24 (6.2)/53 (13.7)	22 (42.3)/12 (23.1)/9 (17.3)/5 (9.6)	0.0079
Residual IASD	37 (8.4)	33 (8.5)	4 (7.7)	0.9241
Deep implantation	18 (4.1)	16 (4.1)	2 (3.8)	0.9508
Pericardial effusion	2 (0.5)	2 (0.5)	0 (0)	0.6082
Device related thrombus	26 (5.9)	24 (6.2)	2 (3.8)	0.4710

Values are n (%).

IASD = iatrogenic atrial septal defect; other abbreviations as in Table 2.

Periprocedural death, device embolization, and any stroke during hospitalization were not observed. Bleeding events including cardiac tamponade occurred in <1% (12 of 1,464) of cases, with no significant difference between both groups.

### Antithrombotic therapy after LAAC

**Figure 1:**
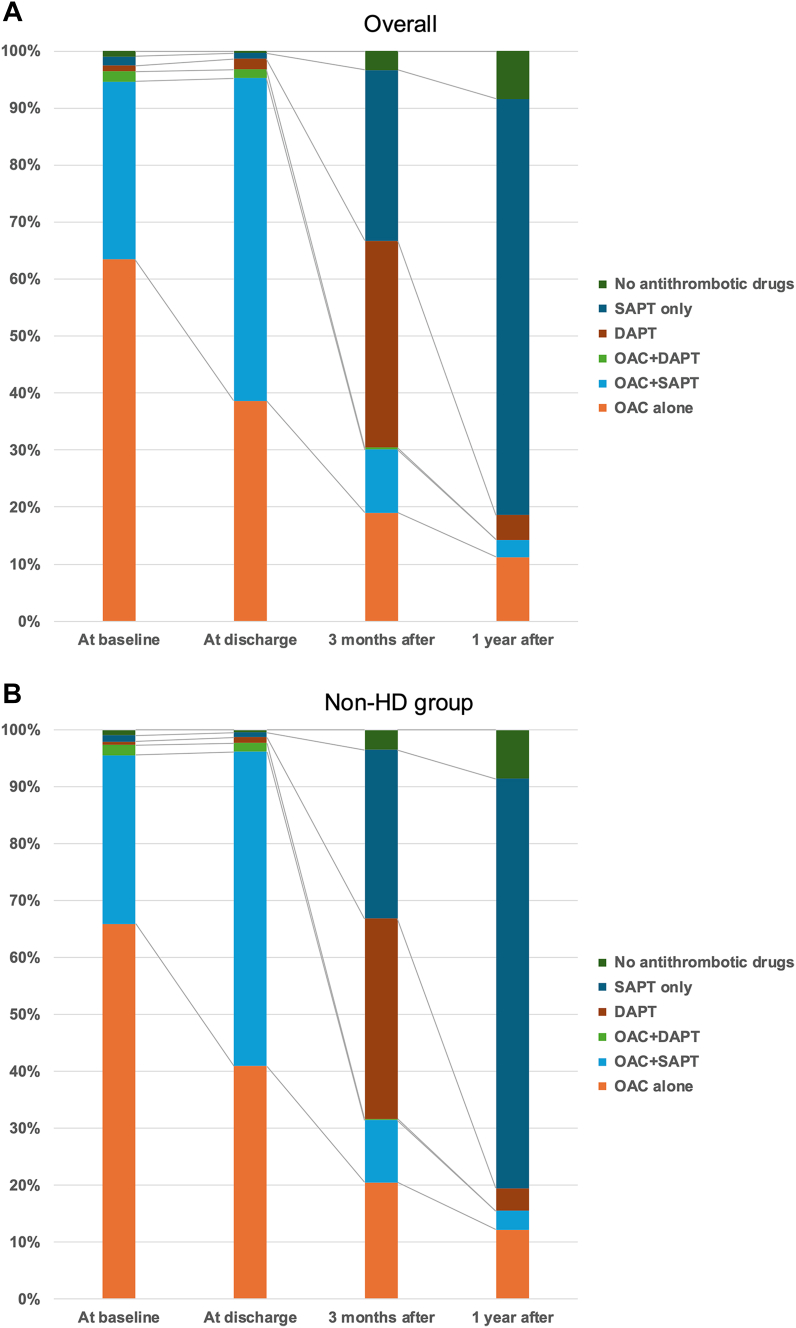

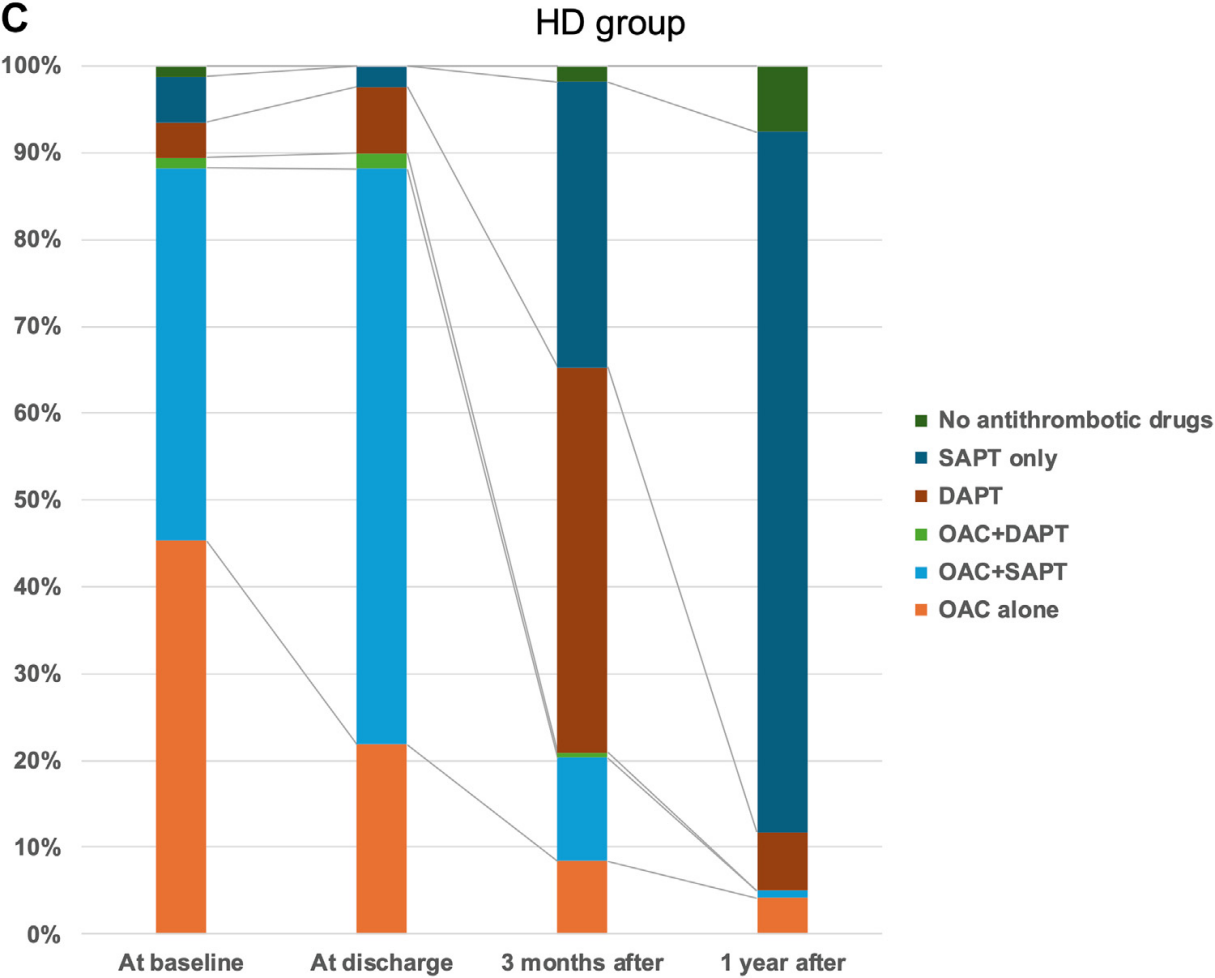
Transition of Antithrombotic Drug Regimen Before and After LAAC (A) Overall cohort, (B) nonhemodialysis (HD) group, (C) HD group. At baseline, 11% of cases in the HD group were not on anticoagulant therapy, but instead, the percentage of cases on antiplatelet therapy was higher compared with the non-HD group. At short-term and 1 year, 95% of cases in the HD group had successfully discontinued anticoagulant therapy. DAPT = dual antiplatelet therapy; LAAC = left atrial appendage closure; OAC = oral anticoagulant; SAPT = single antiplatelet therapy.

The antithrombotic therapy following LAAC was depicted in Figures 1A to 1C. At baseline, 96.4% (1,386 of 1,437) of all patients were anticoagulated, but 10.6% (18 of 170) of patients in the HD group were not anticoagulated, which was a higher rate than in the non-HD group. The percentage of cases using OAC alone was overwhelmingly higher in the non-HD group (65.9% (835 of 1,267) in the non-HD group vs 45.3% (77 of 170) in the HD group; *P* < 0.01), while the proportion of cases using any antiplatelet therapy concomitantly was higher in the HD group (31.5% (399 of 1,267) in the non-HD group vs 44.1% (75 of 170) in the HD group; *P* < 0.01). Obviously, the OAC used in the HD group was warfarin in almost all cases. At discharge, antithrombotic therapy combining OAC and APT was administered in around 60% (HD group; 67.6% [115 of 170], non-HD group; 57.2% [725 of 1,267]) of cases in both groups.

Around 30% (426 of 1,391) of all cases were still taking OAC 3 months after LAAC. The proportion was significantly lower in the HD group, with a higher proportion instead receiving DAPT. Furthermore, only 5.0% (6 of 119) of patients in the HD group were on OAC 1 year after LAAC, a significantly lower rate compared with 15.5% (140 of 904) in the non-HD group (*P <* 0.01).

### Imaging study follow-up

Imaging follow-up at short-term and 1 year were performed in 95.7% (1,391 of 1,453) and 87.4% (978 of 1,119) of all cases, respectively. Among them, the proportion that underwent TEE was 66.7% (953 of 1,428) and 42.0% (440 of 1,047), respectively (Table 3).

In short-term follow-up, DRT was observed in only 1.6% (15 of 918) of all cases. Although there was no significant difference, the proportion of peri-device leakage of 3 mm or more was 5.7% (53 of 926), with a tendency to be lower in the HD group. Additionally, there were no significant differences in other TEE findings between both groups.

In the 1-year follow-up, intradevice thrombosis was more advanced in the non-HD group, but other adverse findings including DRT and peridevice leakage were similarly less frequent compared with the short-term follow-up, and there was no significant difference between both groups.

### Clinical outcome follow-up

**Figure 2:**
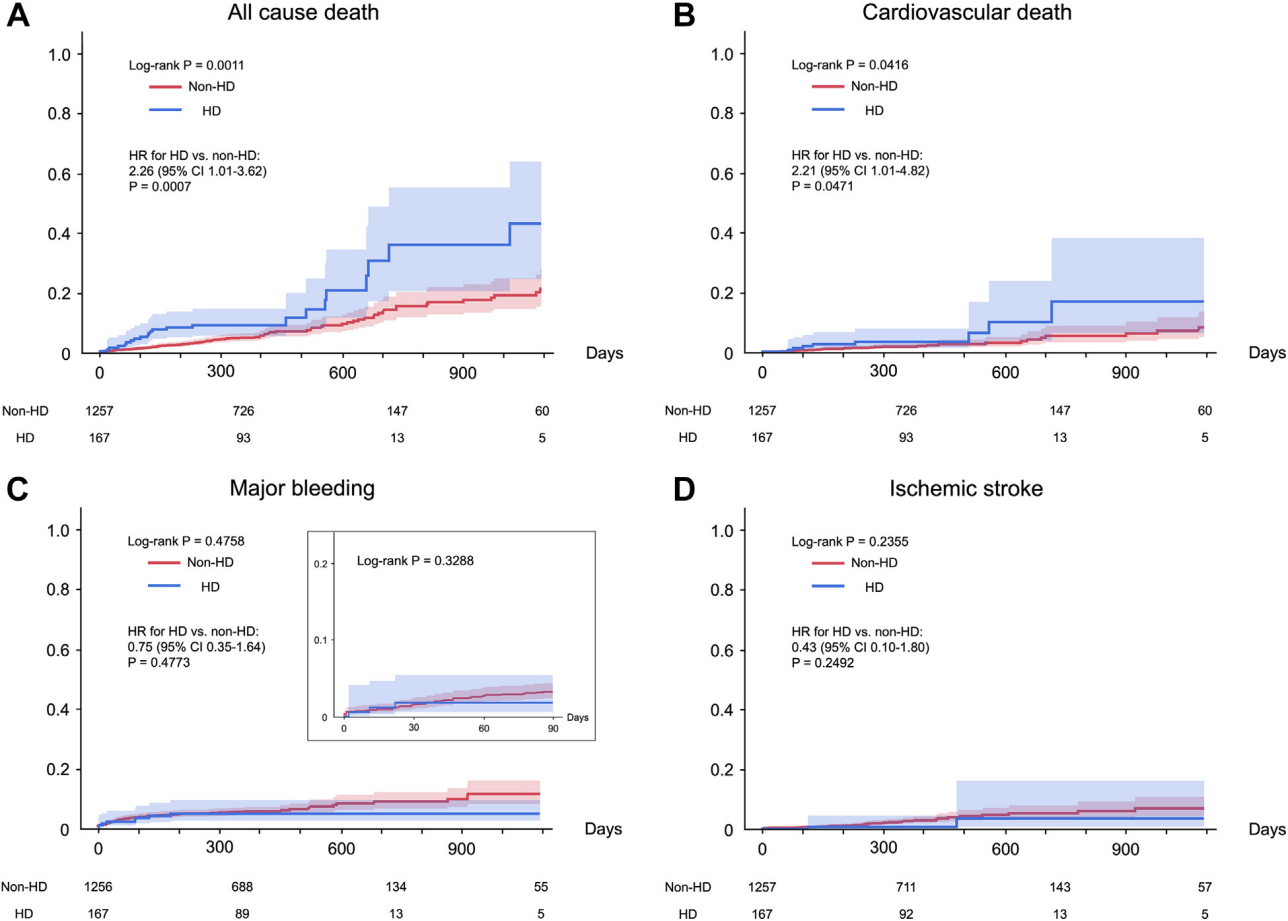
Kaplan-Meier Curve for Adverse Events Following LAAC (A) All-cause death, (B) cardiovascular death, (C) major bleeding, and (D) ischemic stroke. All-cause mortality and cardiovascular mortality were higher in the HD group compared with the non-HD group. Nevertheless, major bleeding and ischemic stroke were comparable between groups, and device-related thrombosis (DRT) tended to be lower in the HD group. Abbreviations as in Figure 1.

The median follow-up period for the overall cohort was 367 days (Q1-Q3: 242-422 days) and not significantly different between groups. The all-cause mortality rate at 1 year was 5.1% overall, with the rate being higher in the HD group at 8.8% compared with 4.6% in the non-HD group. All-cause mortality and cardiovascular mortality were higher in the HD group (Figures 2A and 2B). On the other hand, there was no significant difference in the cumulative incidence of major bleeding or ischemic stroke (Figures 2C and 2D). Even when limited to the period from the procedure until the discontinuation of anticoagulant therapy, there were no particularly high rates of bleeding events in the HD group. The ischemic event rate following LAAC tended to be lower in the HD group at 1.1% (95% CI: 0.3%-1.9%) per 100 patient-years, compared with 2.6% (95% CI: 2.1%-3.0%) per 100 patient-years in the non-HD group.

Additionally, the incidence of DRT was not significantly different between both groups and tended to be lower in the HD groups, with an HR of 0.35 (95% CI: 0.09-1.45; *P =* 0.15).

## Discussion

In this study, we investigated the safety and efficacy of LAAC for HD patients with NVAF using a large-scale cohort in Japan. The main findings are as follows (Central Illustration).

**Central Illustration:**
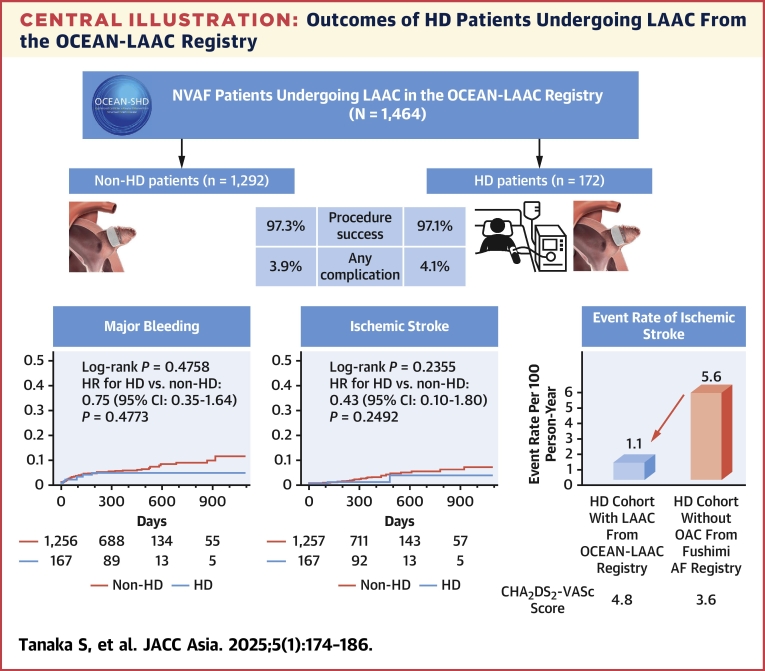
Outcomes of HD Patients Undergoing LAAC From the OCEAN-LAAC Registry Left atrial appendage closure (LAAC) for the hemodialysis (HD) patients demonstrated comparable outcomes to non-HD patients in terms of procedural success and complications. The incidence of adverse events including major bleeding and ischemic stroke was equivalent between the 2 groups. In an indirect comparison between previously reported HD cohort, the HD cohort from the OCEAN (Optimized CathEter vAlvular iNtervention)-LAAC registry had lower incidence of ischemic stroke. NVAF = nonvalvular atrial fibrillation.

First, no significant difference was observed in the safety of the LAAC procedure and in perioperative complications between patients on HD and those who were not. Second, the incidence of DRT, a complication relevant to the long-term outcomes of LAAC, was either comparable between the 2 groups or tended to be lower in the HD group. Last, the incidence of ischemic stroke and hemorrhagic events during the long-term period was statistically comparable between the 2 cohorts. In summary, the presence of HD did not appear to adversely impact the clinical outcomes of the LAA procedure.

### The safety of LAAC for HD patients

Due to various reasons, including comorbid cardiovascular disease, anemia, fluid and electrolyte disturbances, and the differences in metabolism of drugs used in the perioperative period, patients with end-stage renal dysfunction carry higher risks of perioperative morbidity and mortality, regardless of the type of surgery.^11^ LAAC is most commonly performed under general anesthesia because it is recommended to be done with TEE guidance. In this study cohort, all cases were performed under general anesthesia except for 2 cases that were performed under sedation and local anesthesia.

The safety of LAAC has been reported in the PINNACLE FLX trial, which used the newer device before the FLX Pro (the latest device), which is recently launched. The trial reported an implant success rate of 98.8%. In this study, the HD group showed an implant success rate of over 97% (1,424 of 1,464), despite the previous device being used in about 40% (539 of 1,464) of cases, and this success rate was comparable to that of the non-HD group. Although HD patients have generally a high perioperative complication rate, LAAC itself is minimally invasive, using only a transfemoral venous approach, and TEE guidance provides a safer procedure. Furthermore, it is already known that intraoperative complications such as pericardial effusion and bleeding at puncture site are significantly lower with the newer device or the latest device than with the previous device.^12^ Perioperative complications are expected to decrease further in the future even in the HD cohort using these latest devices.

### Antithrombotic therapy following LAAC

Patients with chronic kidney disease, especially those on maintenance HD, have a high incidence of cardiovascular events, many of which are associated with atherosclerosis and arteriosclerosis.^13^^,^^14^ In most cases, APT is needed for secondary prophylaxis. In this study, more than 50% (91 of 170) of HD patients were taking antiplatelet agents at baseline for some reasons, compared with approximately 30% (421 of 1,267) of non-HD patients.

On the other hand, in Japan, anticoagulation therapy for HD patients is generally contraindicated and limited to warfarin. Therefore, in this study, warfarin was used for anticoagulation in almost all HD patients, and it was also newly introduced after LAAC. In addition, according to the protocols of previous randomized control trials,^1^^,^^2^ it is recommended to add APT after LAAC during short-term follow-up. In the present study, nearly 80% (132 of 172) of HD groups were taking APT at the time of discharge.

The biggest concern is the increase in bleeding events from a few weeks before LAAC to short-term follow-up period. Against our anxiety, the rate of bleeding during the period in the HD group was as low as those of non-HD group. Furthermore, in the real-world data in the United States, it has been reported that there were fewer bleeding events without an increase in embolic events even without the addition of APT.^15^ This suggests that the protocol of not adding APT following LAAC may become mainstream in the near future. Furthermore, protocols for promptly discontinuing anticoagulant therapy and switching to DAPT after the procedure are being reported, and their usefulness is starting to be demonstrated.^16^ These changes would further reduce the bleeding events after LAAC.

Permanent single APT is a recommended antithrombotic therapy following LAAC. The contribution of single APT to bleeding events in HD patients is known not to be significant,^17^^,^^18^ and it is not necessary to prioritize in the decision-making for LAAC indication.

### The long-term clinical outcomes

Patients on HD generally exhibit a worse prognosis compared with non-HD patients, with a notably higher incidence of cardiovascular diseases such as ischemic heart disease, cerebrovascular disease, and heart failure, in addition to noncardiovascular events like infections and malignant tumors.^19^ Unfortunately, this trend was also evident in the present study, where both all-cause and cardiovascular mortality were significantly higher in the HD group compared with the non-HD group.

Although mean HAS-BLED score was approximately 1 point higher in the HD group than in the non-HD group, there was no significant difference in bleeding events after the LAAC between both groups. Considering that anticoagulant agents contribute to increased bleeding events in HD patients,^7^^,^^20^ not taking warfarin in the chronic phase may help prevent an increased risk of bleeding caused by renal dysfunction. In addition, differences in patient characteristics, such as older age and a higher proportion of patients with a history of bleeding events in the non-HD group, may have contributed to the lack of difference between both groups.

**Figure 3:**
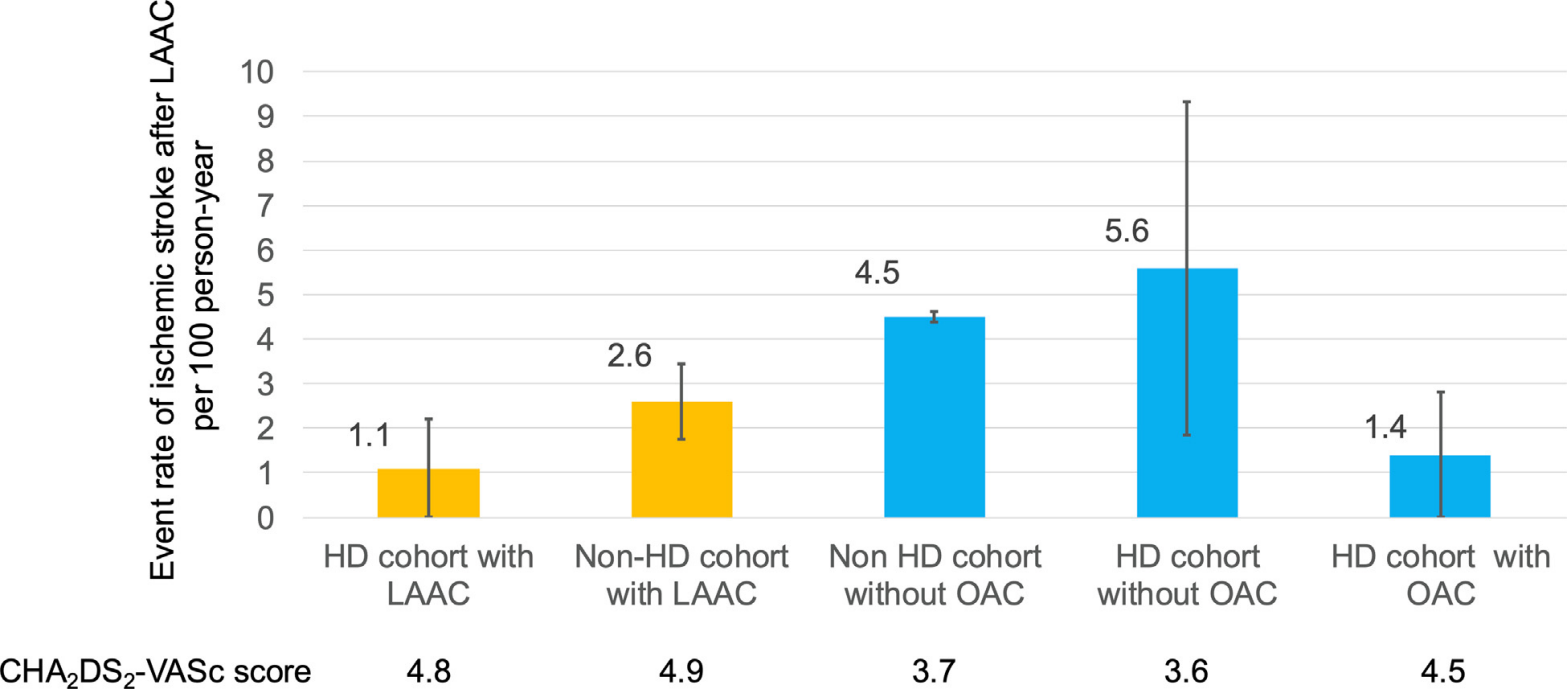
Ischemic Stroke Rates After LAAC Compared to Previous Studies The event rate of ischemic stroke in the HD cohort was higher compared to the non-HD cohort. Left atrial appendage closure (LAAC) in the HD cohort could reduce the event rate of ischemic stroke comparable to the reduction observed in HD patients receiving anticoagulant therapy.^22^^,^^23^ Abbreviations as in Figure 1.

The event rate for ischemic stroke did not differ significantly between both groups. However, as illustrated in Figure 3, the HD patients who underwent LAAC may exhibit a lower incidence of ischemic stroke when indirectly associated with the occurrence of DRT.^21^ Interestingly, the incidence of DRT appeared to be relatively lower in the HD group. This reduction may be partly attributed to the intermittent anticoagulation with heparin administered during dialysis sessions, which could contribute to decreasing the incidence of DRT.

### The indication of LAAC for HD patients and future concerns

Because the benefit of anticoagulation for HD patients with NVAF is inconsistent and often contraindicated, the aim for LAAC in this cohort is not to be an alternative to permanent anticoagulation, but to reduce the risk of cardiogenic embolism. Although not directly comparable in terms of efficacy, the incidence of ischemic stroke was lower in previous reports^22^^,^^23^ of ischemic stroke in HD patients with NVAF (Figure 3).

Additionally, the latest device, currently available on the market, incorporates a fluoropolymer-coated fabric membrane designed to enhance thrombo-resistance and promote endothelialization,^24^ which are expected to enable shorter antithrombotic regimens. In animal experiments, there was reduced incidence of DRT and tissue inflammation observed without the administration of antithrombotic agents.^25^ It is hoped that further clinical trials utilizing these novel devices will accumulate evidence and enhance recognition of the usefulness of LAAC in HD patients.

### Study limitations

First, because this is a multicenter observational study, each facility has some discretion. Therefore, data variability is possible, and under-reporting of events cannot be ruled out. Second, baseline characteristics such as the number of each group, the HAS-BLED score, and the percentage of patients with a history of bleeding were different between both groups. The HAS-BLED score was higher in the HD groups and the percentage of patients with a history of bleeding was higher in the non-HD group. Potential differences in patient populations may have influenced the results. However, this reflects real-world data, so we opted to make a direct comparison. Third, follow-up imaging data was limited to TEE and did not include contrast-enhanced computed tomography. Although adverse events such as DRT can be detected regardless of the modality, the analysis of anatomical details, such as peridevice leakage and deep implantation, may differ when using both contrast-enhanced computed tomography and TEE. Fourth, the observational period was relatively short. Long-term outcomes are a very important factor in LAAC. Further long-term observation will be important in evaluating its usefulness. Finally, only a single type of LAAC device was used in this study. It should be noted that other LAAC devices may yield different results compared to the present study.

## Conclusions

Despite the aforementioned limitations, this study suggests that LAAC is a safe procedure for HD patients, yielding outcomes comparable to those observed in non-HD patients. However, further research is necessary to determine the efficacy of LAAC in preventing stroke specifically in the HD population.

## Funding Support and Author Disclosures

The OCEAN-LAAC registry, which is part of the OCEAN-SHD registry, is supported by Edwards Lifesciences, Medtronic, Boston Scientific, Abbott Medical, and Daiichi-Sankyo Company. Drs Nakashima, Yamamoto, Asami, Hachinohe, Ueno, and Kubo are clinical proctors for Boston Scientific. Dr Yamamoto has received lecture fees from Daiichi-Sankyo and Boston Scientific. Dr Saji has received lecture fees from Daiichi-Sankyo and Abbott Medical Japan. All other authors have reported that they have no relationships relevant to the contents of this paper to disclose.

## References

[1] Reddy V.Y., Sievert H., Halperin J. (2014). Percutaneous left atrial appendage closure vs warfarin for atrial fibrillation: a randomized clinical trial. JAMA.

[2] Holmes D.R., Kar S., Price M.J. (2014). Prospective randomized evaluation of the Watchman Left Atrial Appendage Closure device in patients with atrial fibrillation versus long-term warfarin therapy: the PREVAIL trial. J Am Coll Cardiol.

[3] Aonuma K., Yamasaki H., Nakamura M. (2018). Percutaneous WATCHMAN left atrial appendage closure for Japanese patients with nonvalvular atrial fibrillation at increased risk of thromboembolism- first results from the SALUTE Trial. Circ J.

[4] Asami M., Naganuma T., Ohno Y. (2023). Initial Japanese multicenter experience and age-related outcomes following left atrial appendage closure: the OCEAN-LAAC registry. JACC Asia.

[5] Kar S., Doshi S.K., Sadhu A. (2021). Primary outcome evaluation of a next-generation left atrial appendage closure device: results from the PINNACLE FLX trial. Circulation.

[6] Toida T., Sato Y., Nakagawa H. (2016). Risk of Cerebral Infarction in Japanese Hemodialysis Patients: Miyazaki Dialysis Cohort Study (MID study). Kidney Blood Press Res.

[7] Kuno T., Takagi H., Ando T. (2020). Oral anticoagulation for patients with atrial fibrillation on long-term hemodialysis. J Am Coll Cardiol.

[8] Mehran R., Rao S.V., Bhatt D.L. (2011). Standardized bleeding definitions for cardiovascular clinical trials: a consensus report from the Bleeding Academic Research Consortium. Circulation.

[9] Tzikas A., Holmes D.R., Gafoor S. (2017). Percutaneous left atrial appendage occlusion: the Munich consensus document on definitions, endpoints, and data collection requirements for clinical studies. Europace.

[10] Boersma L.V., Schmidt B., Betts T.R. (2016). Implant success and safety of left atrial appendage closure with the WATCHMAN device: periprocedural outcomes from the EWOLUTION registry. Eur Heart J.

[11] Kanda H., Hirasaki Y., Iida T. (2017). Perioperative management of patients with end-stage renal disease. J Cardiothorac Vasc Anesth.

[12] Nakashima M., Yamamoto M., Sago M. (2024). Comparative data of procedural and midterm outcomes in patients who underwent percutaneous left atrial appendage closure between the WATCHMAN FLX and WATCHMAN 2.5 devices - insight from the OCEAN-LAAC Registry. Circ J.

[13] Ninomiya T., Kiyohara Y., Kubo M. (2005). Chronic kidney disease and cardiovascular disease in a general Japanese population: the Hisayama Study. Kidney Int.

[14] Chirakarnjanakorn S., Navaneethan S.D., Francis G.S., Tang W.H. (2017). Cardiovascular impact in patients undergoing maintenance hemodialysis: clinical management considerations. Int J Cardiol.

[15] Freeman J.V., Higgins A.Y., Wang Y. (2022). Antithrombotic therapy after left atrial appendage occlusion in patients with atrial fibrillation. J Am Coll Cardiol.

[16] Berti S., De Caterina A.R., Grasso C. (2023). Periprocedural outcome in patients undergoing left atrial appendage occlusion with the Watchman FLX device: The ITALIAN-FLX registry. Front Cardiovasc Med.

[17] Chen Z.W., Wu C.K., Yang Y.H. (2019). Efficacy of antiplatelet agent usage for primary and secondary prevention in dialysis patients: a nationwide data survey and propensity analysis. Cardiovasc Drugs Ther.

[18] Chen C.Y., Lee K.T., Lee C.T., Lai W.T., Huang Y.B. (2014). Effectiveness and safety of antiplatelet in stroke patients with end-stage renal disease undergoing dialysis. Int J Stroke.

[19] Vogelzang J.L., van Stralen K.J., Noordzij M. (2015). Mortality from infections and malignancies in patients treated with renal replacement therapy: data from the ERA-EDTA registry. Nephrol Dial Transplant.

[20] Olesen J.B., Lip G.Y., Kamper A.L. (2012). Stroke and bleeding in atrial fibrillation with chronic kidney disease. N Engl J Med.

[21] Simard T., Jung R.G., Lehenbauer K. (2021). Predictors of device-related thrombus following percutaneous left atrial appendage occlusion. J Am Coll Cardiol.

[22] Friberg L., Rosenqvist M., Lip G.Y. (2012). Evaluation of risk stratification schemes for ischaemic stroke and bleeding in 182 678 patients with atrial fibrillation: the Swedish Atrial Fibrillation cohort study. Eur Heart J.

[23] Yamashita Y., Takagi D., Hamatani Y. (2016). Clinical characteristics and outcomes of dialysis patients with atrial fibrillation: the Fushimi AF Registry. Heart Vessels.

[24] Nielsen-Kudsk J.E., Kramer A., Andersen A., Kim W.Y., Korsholm K. (2024). First-in-human left atrial appendage closure using the WATCHMAN FLX Pro device: a case report. Eur Heart J Case Rep.

[25] Saliba W.I., Kawai K., Sato Y. (2023). Enhanced thromboresistance and endothelialization of a novel fluoropolymer-coated left atrial appendage closure device. JACC Clin Electrophysiol.

